# Supplementary figures and images for: Combination adjuvants drive long-lived plastic Th17 cells that convert to multi-functional Th1 cells and protect mice against fungal infection
Source: mBio. 2026 Jun 11;17(7):e00505-26. doi: 10.1128/mbio.00505-26 (PMC13343890; doi:10.1128/mbio.00505-26)

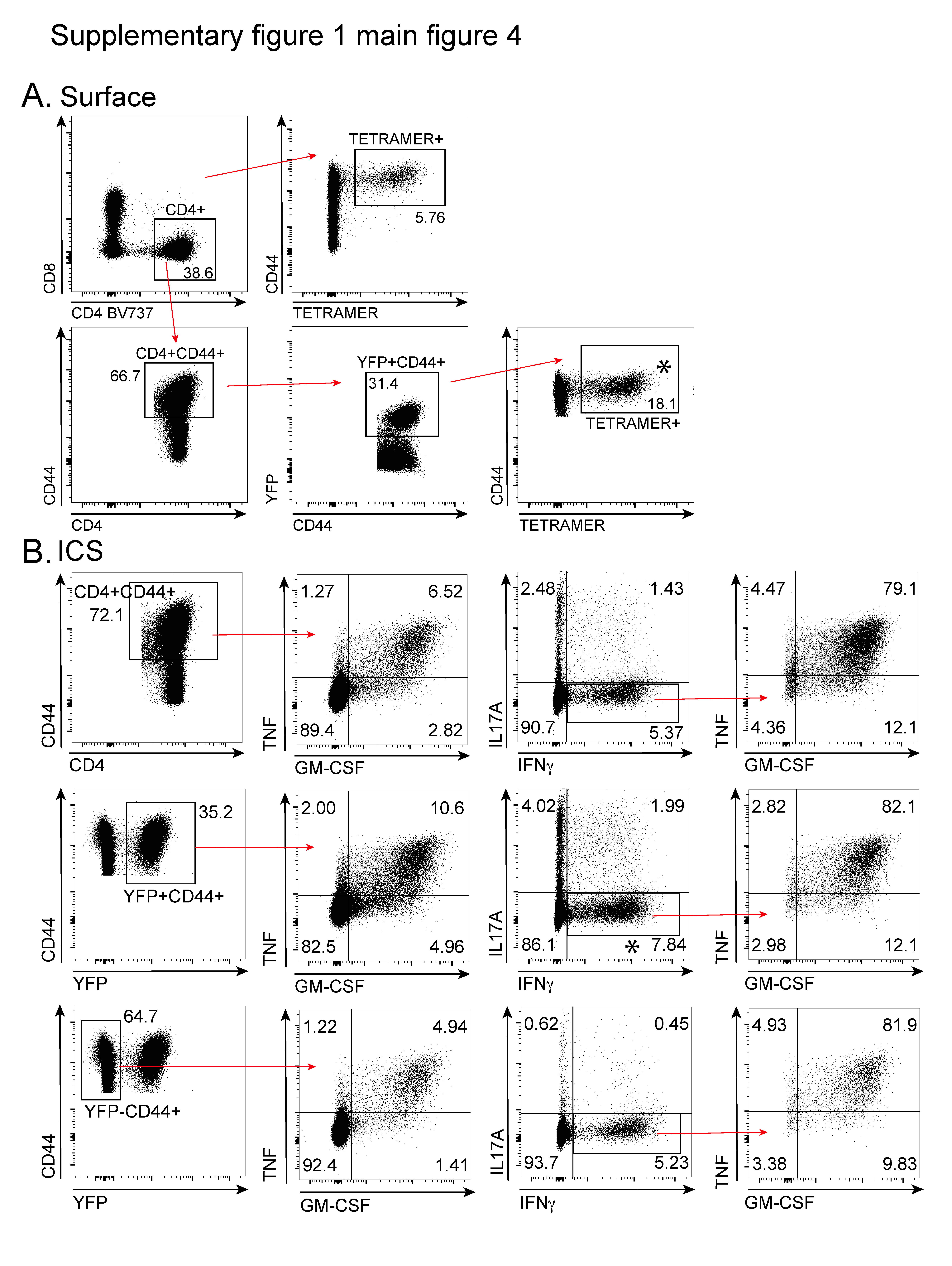

Supplement: Figure S1 — Polyfunctional T cells in IL-17 reporter mice vaccinated with GCP-Bl-Eng2 + GLA. [file mbio.00505-26-s0001.tiff]
