# Supplementary material for: Combination adjuvants drive long-lived plastic Th17 cells that convert to multi-functional Th1 cells and protect mice against fungal infection
Source: mBio. 2026 Jun 11;17(7):e00505-26. doi: 10.1128/mbio.00505-26 (PMC13343890; doi:10.1128/mbio.00505-26)
Supplement: Legends — Supplemental figure legends. [file mbio.00505-26-s0004.pdf]

SUPPLEMENTAL FIGURE LEGENDS

SFig. 1 for main figure 4: Polyfunctional T cells in IL-17 reporter mice vaccinated with GCP-*Bl*-Eng2+GLA. Mice were vaccinated with GCP-*Bl*-Eng2+GLA twice and challenged 3 months post-vaccination. The frequencies of tetramer$^+$ T cells were determined from total CD4$^+$ T cells and CD4$^+$ CD44$^+$ eYFP$^+$ T cells (**A**). The frequencies of cytokine (GM-CSF, TNF and IFN-γ) producing T cells were determined from CD4$^+$ CD44$^+$ T cells and the frequencies of GM-CSF and TNF producing T cells from IFN-γ^+ T cells (**B**). *p<0.05, vs. all other groups, Anova test.

SFig. 2 for main figure 5: Number of tetramer$^+$, eYFP$^+$ and cytokine producing T cells in unchallenged and challenged reporter mice. eYFP reporter mice were vaccinated with GCP-Eng2+GLA thrice two weeks apart and rested for 3 months before challenge. Lung T cells were analyzed in challenged reporter mice 6 days post-infection and in splenocytes from unchallenged reporter mice. The number of tetramer$^+$ T cells (**A**), eYFP$^+$ T cells (**B**), eYFP$^+$ IFN-γ^+ T cells (**C**), IL-17$^+$ T cells (**D**), IFN-γ^+ T cells (**E**) and ex-Th17$^+$Th1 cells (**F**) were enumerated. *p<0.05, vs. all other groups, Anova test.

SFig. 3 for main figure 6: Properties of donor T cells. Donor T cells were analyzed before adoptive transfer and lung CFU of the donors was enumerated 4 days post-infection. Donor mice were vaccinated with GCP-*Bl*-Eng2+GLA twice and rested for 3 months. Lung T cells were stained for tetramer (**A**) and cytokines (**B**) and analyzed by FACS. Lung CFU were plated from the same mice (**C**). *p<0.05, 2-tailed, Mann-Whitney U test.
